# Impact of Terminal Heat and Combined Heat-Drought Stress on Plant Growth, Yield, Grain Size, and Nutritional Quality in Chickpea (*Cicer arietinum* L.)

**DOI:** 10.3390/plants12213726

**Published:** 2023-10-30

**Authors:** Aouatif Benali, Noureddine El Haddad, Somanagouda B. Patil, Aakash Goyal, Kamal Hejjaoui, Adil El Baouchi, Fatima Gaboun, Mouna Taghouti, Mohammed Ouhssine, Shiv Kumar

**Affiliations:** 1 Laboratory of Agro-Physiology, Biotechnology, Environment and Quality, Department of Biology, Faculty of Sciences, IbnTofail University, Kenitra 14000, Morocco; mohammed.ouhssine@uit.ac.ma; 2 National Institute of Agricultural Research (INRA), Rabat-Instituts, Rue Hafiane Cherkaoui, Rabat 10101, Morocco; 3 International Center for Agricultural Research in the Dry Areas (ICARDA), Rabat-Instituts, Rue Hafiane Cherkaoui, Rabat 10101, Morocco; n.el-haddad@cgiar.org (N.E.H.); sbpatil84@gmail.com (S.B.P.); akgroyal@gmail.com (A.G.); 4 AgroBioSciences, Mohammed VI Polytechnic University, Ben Guerir 43150, Morocco; kamal.hejjaoui@um6p.ma (K.H.);; 5 International Center for Agricultural Research in the Dry Areas (ICARDA), New Delhi 110012, India

**Keywords:** chickpea, drought stress, heat stress, nutritional quality, stress selection indices, micronutrient deficiency

## Abstract

Chickpea is the third most consumed pulse and provides a kit of essential nutrients for an exponential population. High temperatures and drought stress are two major abiotic stresses that cause serious effects on chickpea growth and development. The comprehension of abiotic stresses’ impact on chickpea productivity and nutritional quality will permit the selection of promising genotypes. The current study aimed to assess the impact of heat and drought stresses on plant growth, grain yield and its components, grain size, and nutritional quality in chickpea. For this purpose, 43 international chickpea genotypes were evaluated under normal, heat, and combined heat-drought stress conditions. The findings revealed a significant decrease of over 50% in plant height, biological yield, and seed yield under both stress conditions. Grain size and hundred-seed weight were the most heritable traits under normal, heat, and combined heat-drought stress. Proteins were accumulated under both stresses, evolving from 20.26% for normal conditions to 22.19% for heat stress and to 21.94% for combined heat-drought stress. For minerals, significant variation between treatments was observed for Mn, Mg, and Na. Our results also showed a significant impact of genotype and genotype-environment interaction factors only on K content. Using selection indices, 22 genotypes were identified as highly tolerant to the combined heat-drought stress, while eleven genotypes were heat-tolerant. Mineral profile analysis according to the contrasting tolerance clusters revealed decreased potassium content in susceptible genotypes, indicating genetic potential in the studied chickpea collection, ensuring tolerance to both stresses while maintaining good grain quality.

## 1. Introduction

Hidden hunger, or micronutrient deficiency, is the main burden of malnutrition [1], to the extent that one in three people in the world suffers from it [2]. Low content of essential minerals and their low bioavailability are among the serious causes of this burden, given that mineral nutrients for their various functionalities and potentials in the body’s metabolism are essential for the maintenance of life and their deficiency can have serious and long-term consequences on human health [3,4]. The consequences of this nutritional deficit can range from poor health with low productivity up to death by increasing susceptibility to infections. Recent studies have shown positive correlations between the fatal prevalence of COVID-19 and a high level of malnutrition [5,6]. Furthermore, its occurrence is even more prominent among certain categories and human life stages. Pregnant women and preschool children are the most affected. Global reports indicate that 162 million young children are still suffering from chronic undernutrition. In fact, prenatal malnutrition causes in-utero fetal growth problems and low birth weight during the first 1000 days, causing a risk of permanent cognitive impairment and physical disability in the child [7,8,9,10].

Chickpea (*Cicer arietinum* L.) is a self-pollinated pulse crop that originated in the Fertile Crescent about 9500 years ago. In terms of production, it ranked third after beans and peas, accounting for 20% of the world’s pulse production. Currently, it is grown in temperate and semiarid climates in about 50 countries, with India as the largest producer, contributing 65% to the global production [11,12]. Recently, chickpea has emerged as the next functional food for human consumption as it is an affordable source of high-quality protein and health-promoting fatty acids, fibers, and micronutrients [13,14]. On average, chickpea seeds contain 61–68% carbohydrates, 20–26% protein, 5–6% fat, 3–8% resistant starch, and 3–5% crude fiber by weight [15,16,17]. Chickpeas also contain significant amounts of potassium, phosphorus, calcium, iron, and zinc. The consumption of 100 g of cooked chickpeas can provide up to 44% of the recommended daily allowance of some minerals for adults and much more for children between 1–3 years old [18,19]. This nutritional composition imparts to it many preventive and therapeutic properties for ailments such as type 2 diabetes and cancer [20].

In the Mediterranean climate, chickpeas are planted during the winter and spring seasons [21] and are considered a thermosensitive crop. Temperatures exceeding 35 °C can cause serious damage to its growth and yield [22,23]. Although chickpea is considered a drought-tolerant crop [24], late plating in the spring season makes it susceptible to the effect of terminal drought stress, which is an extended stress leading to an incessant water deficit condition, unlike a temporary lack of water overcome by precipitation [25].

Terminal drought is often accompanied by temperature rises that manifest negatively on the reproductive phase and pod filling [26]. resulting in severe plant yield and yield traits losses of up to 100% in susceptible chickpea genotypes [21,27]. This plant agronomic decline under individual and combined stress is mainly due to poor pod formation because of the deterioration of pollen grains’ viability and their germination on the stigma and pollen tube growth [28,29,30]. These abiotic stresses damaged membranes and decreased cellular oxidizing ability, stomatal conductance, PSII function, and leaf chlorophyll content. These effects were pronounced in heat and drought-sensitive chickpea genotypes [31].

Few recent studies have reported contrasted drought and heat stress effects on the nutritional qualities of various legumes, including chickpea [27,28,32], lentil [33,34,35], and bean [36]. Nevertheless, more investigations on the influence of these stress forms on chickpea yield and quality parameters are required. The objective of this study was to (i) compare the yield, agronomic performance, grain size, protein content, and mineral profile of an international collection of chickpeas under normal, heat, and combined heat-drought stress conditions and (ii) identify a new source of tolerance in chickpeas to heat and drought stresses while maintaining a high nutritional quality.

## 2. Results

### 2.1. Plant Growth, Seed Yield and Grain Size under Normal and Stress Conditions

Analysis of variance indicated significant variability for all phenotypic parameters under normal and stress conditions (Tables S1 and S2). Both heat stress and combined heat-drought stress severely affected plant height (PH) and grain yield (GY). Further, first pod height (FPH), biological yield (BY), PH, and GY decreased approximately by 50% compared to normal conditions. In contrast, no significant difference between the three treatments was observed for harvest index (HI). However, HI declined by 6.46% under heat stress and by 2.32% under combined heat-drought stress. The influence of the genotype factor was highly significant (<0.001) for almost all traits; however, the G × E interaction did not show a significant effect for any of the measured parameters except for PH. In normal sowing of the second season (N2), the heritabilities of PH and BY were estimated at H^2^ = 0.71 and H^2^ = 0.39, while their heritabilities were decreased to H^2^ = 0.37 for PH and H^2^ = 0.24 for BY due to heat stress, and H^2^ = 0.38 for PH and H^2^ = 0.17 for BY under combined heat-drought stress. Heritability of GY was H^2^ = 0.40 under N2, H^2^ = 0.57 under heat stress, and H^2^ = 0.57 under the combined stress conditions. Grain size parameters and hundred-seed weight (HSW) were significantly affected by the combined heat-drought stress and decreased by 9% for HSW, 5% for area, 4% for width, 2% for perimeter, and 3% for length. The effects of genotype and GxE interaction were highly significant (<0.001) for grain size parameters and HSW (Tables S1 and S3). High heritability was observed for these parameters independently of the year or the applied stress (Table S3).

### 2.2. Effect of Heat and Combined Heat-Drought Stress on Nutritional Quality

Our results revealed that genotype, year, and treatment factors had a significant effect (*p* < 0.01) on protein content (Tables S1 and S2). Heat and combined heat-drought stresses have a significant cumulative effect on protein content, evolving to 9.45% for heat stress and 8.1% for combined stress as compared to normal conditions (N2).

Our results indicated no significant differences between the three treatments for all minerals except for manganese, magnesium, and sodium (*p* < 0.001). Compared to normal conditions, manganese increased by 15.8% under heat stress and by 12.11% due to the combined heat-drought stress. In addition, magnesium content increased by 9.8% due to heat stress and 3.5% because of combined heat and drought stress. Sodium was increased by 18.7% under heat stress and by 20.8% in combined stress conditions. Iron decreased by 9.7% under heat stress and increased slightly under combined heat-drought stress (Table S3). The genotype factor had a significant effect (*p* < 0.01) only on selenium and potassium, while the GxE interaction was significant for zinc and potassium (*p* < 0.001) (Table S1).

Our findings demonstrated a highly significant effect of year (*p* < 0.001) on all minerals, with the exception of selenium and potassium, when comparing the results between the two normal experiments. In addition, the genotype factor was significant for potassium and magnesium (*p* < 0.01). The genotype x year interaction was significant for selenium, potassium, and magnesium (*p* < 0.01) (Table S2). Heritability of quality parameters was generally low to medium across the four environments. Potassium was the only mineral with a considerable decrease in heritability under the two stress conditions (Table S3).

### 2.3. Correlations among Plant Morphology, Grain Size and Nutritional Quality Parameters

Correlation analysis revealed that BY and GY had a positive correlation (*p* < 0.01) with HI, while PH and FPH were positively correlated under heat stress and combined heat-drought stress. In addition, BY was positively correlated with PH (*p* < 0.01) under combined heat-drought stress. GY showed a significant negative correlation (*p* < 0.01) with FPH under combined stress but was not significant under heat stress conditions (Table S4). Under both normal treatments (N1 and N2), GY was highly positively correlated with HI and BY (Table S5). All four parameters of grain morphology (area, perimeter, length, and width) showed a significant positive correlation (*p* < 0.01) with HSW under normal conditions, heat stress, and combined heat-drought stress. Under heat stress, area, perimeter, and length had a negative correlation (*p* < 0.05) with HI.

For minerals, selenium (Se) and magnesium (Mg) demonstrated significant negative correlations with BY and GY under the combined heat-drought stress. Further, Se was negatively correlated with GY, BY, and protein content and positively correlated with HSW, area, and sodium (Na) (*p* < 0.05) under the combined stress conditions. Under heat stress, iron (Fe) was significantly negatively correlated (*p* < 0.05) with HI and potassium (K); however, copper (Cu) had a positive correlation (*p* < 0.01) with protein content, K, and manganese (Mn). Na was negatively correlated (*p* < 0.05) with HI and positively correlated (*p* < 0.01) with Mn. Under normal conditions (N1), our results revealed a positive correlation of K and Cu with HSW and grain size parameters. As to normal planting (N2), Se correlated positively (*p* < 0.05) with PH, similarly for K with FPH and Na with Mg. However, Na was negatively correlated with HSW and grain size parameters. In addition, a positive correlation was found between calcium (Ca), K, Mg, Mn, and Cu under normal conditions (N1), while Mg and Ca were positively correlated with HI. Our results also showed a high negative correlation between Ca and protein content (*p* < 0.05), whereas Se was negatively correlated with grain length under normal conditions (N1). Under both normal conditions, zinc (Zn) was positively correlated (*p* < 0.01) with Fe.

### 2.4. Drought and Heat Tolerance Selection

#### 2.4.1. Correlation Analysis

Pearson correlation coefficients were performed to identify associations between yield under stress conditions (Ys), yield under optimal conditions (Yp), and stress indices for heat and combined heat-drought stresses. Under both stress conditions, Ys and Yp were significantly positively (*p* < 0.01) correlated with the majority of the stress indices, except for TOL for Ys and SSI and YSI for Yp. A significant negative (*p* < 0.01) correlation was detected between Ys and SSI (Table 1). In addition, STI and GMP had a high positive correlation of 0.01% with Ys, Yp, MP, HARM, and YSI, whereas they exhibited a negative correlation with SSI under both stress conditions.

#### 2.4.2. PCA Analysis and Hierarchical Clustering

Principal component analysis (PCA) was conducted to investigate the relationship between traits in the examined chickpea genotypes under the two stress conditions. Under heat stress treatment, the first two axes (PC1: 67.95% and PC2: 31.09%) explained 99.04% of the total variation, while PC3 showed 0.75% of the total variation (Table 2). For the combined heat-drought stress conditions, the first three axes (PC1: 69.39%, PC2: 62.68% and PC3: 0.68%) explained 99.07% of the total variability. In addition, the results of PCA revealed that Ys, GMP, MP, HARM, STI, Yp and YSI were the most important traits contributing to PC, while PC2 was highly correlated (*p* < 0.001) with Yp, TOL, SSI and YSI under both stress conditions (Table S6).

**Figure 1:**
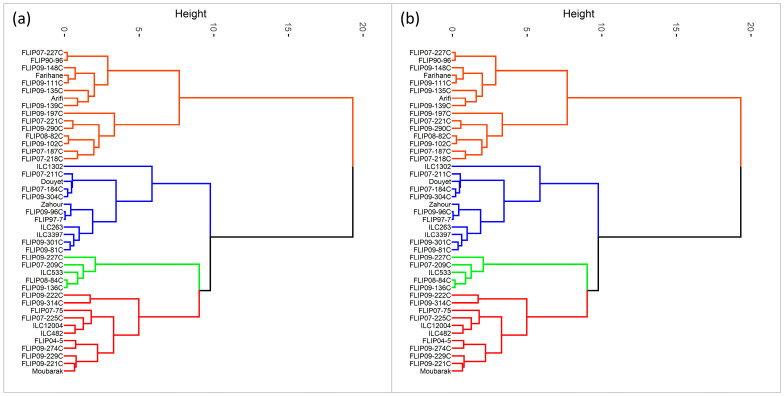
Classification of chickpea genotypes under heat stress conditions (**a**) and combined heat-drought stress conditions (**b**) using the different stress indices (Red: cluster 1, Green: cluster 2, Blue: cluster 3, Orange: cluster 4).

**Figure 2:**
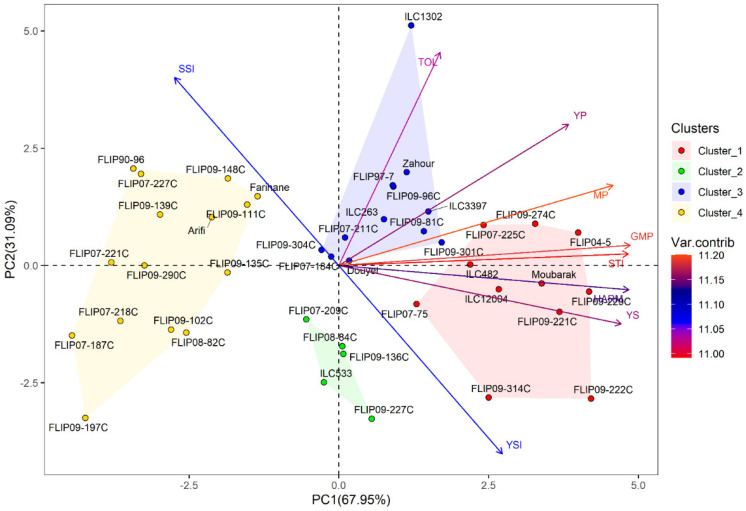
Biplot of the first two principal components PC1 and PC2 of genotypes and stress indices under heat stress conditions at Marchouch during 2016–2017.

Furthermore, hierarchical cluster analysis was performed for both stress conditions, and the genotypes were grouped into four clusters (Figure 1). Under heat stress, cluster 1 represented individuals with the highest Ys, STI, GMP, MP, and HARM (Figure 1a and Table S7) and a very high yield under normal conditions (Yp). This cluster with 11 genotypes can be classified as a heat-tolerant cluster, i.e., FLIP04-5C, FLIP09-229C, and FLIP09-222C. The second cluster had 5 genotypes characterized by a moderately high yield compared to the first cluster with the lowest TOL and SSI and the highest YSI; these genotypes are considered moderately tolerant to heat stress (FLIP09-136C, FLIP07-209C). The third cluster comprised 12 genotypes, i.e., Douyet, FLIP09-301C, and ILC3397, which corresponded to moderately susceptible genotypes with less yield than the antecedent group. Cluster 3 had the highest TOL and Yp and moderate GMP, MP, and SSI. This cluster represented genotypes with high grain yields in optimal conditions. The fourth cluster (15 genotypes) included susceptible genotypes with low yield performance (highest SSI and lowest YSI), i.e., Farihane, FLIP09-111C, and FLIP09-148C. The biplot of PC1 and PC2 of the four clusters and measured traits under heat stress is demonstrated in Figure 2.

**Figure 3:**
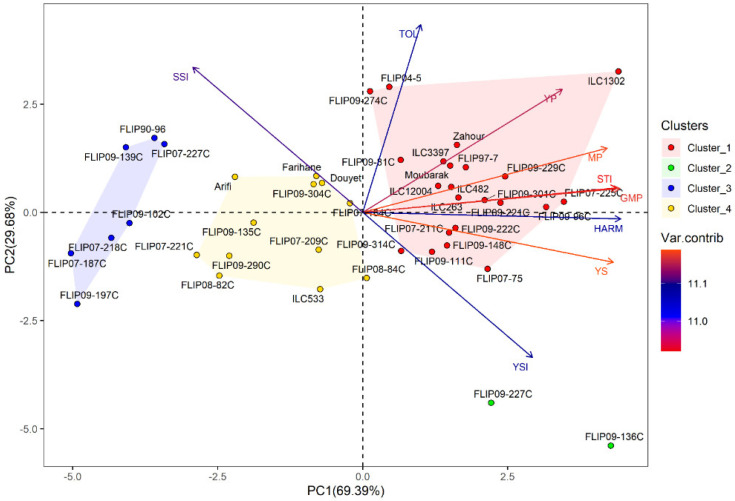
Biplot of the first two principal components PC1 and PC2 of genotypes and stress indices under combined heat-drought stress conditions at Marchouch during 2016–2017.

Under combined heat-drought stress, 22 genotypes were identified as tolerant, i.e., (ILC1302, FLIP07-225C, FLIP09-96C), thanks to their high Ys, STI, GMP, MP, HARM, and Yp (Figure 1b and Table S8). Two genotypes (FLIP09-136C and FLIP09-227C) representing the second cluster had good grain yield under combined heat-drought stress (highest Ys and YSI) and potentially had low yields under optimal conditions, classifying them as moderately tolerant. The third group (12 genotypes) concerned the moderately susceptible genotypes (cluster 4), i.e., FLIP07-184C, FLIP08-84C, Douyet, and lastly the susceptible genotypes corresponding to cluster 3 (7 genotypes), i.e., FLIP07-227C, FLIP90-96, and FLIP09-102C, with the lowest yields (Yp and Ys), STI, GMP, MP, HARM, and YSI, and the highest SSI. The biplot of PC1 and PC2 of the four clusters and traits under combined heat-drought stress conditions is shown in Figure 3.

### 2.5. Plant Growth, Yield, Grain Morphology and Quality under the Identified Clusters

Analysis of trait means under both heat stress and combined heat-drought stress revealed significant differences between the four clusters, especially for GY and HI, where the tolerant group exhibited notably higher values. The maximum grain yield was 89.10 g per plot under heat stress and 112.33 g per plot under combined heat-drought stress (Table 3). HI was 37.56% for tolerant genotypes under heat stress and 38.26% for moderately tolerant genotypes under combined heat-drought stress. The PH of clusters 2 and 3 was significantly higher at *p* < 0.05 than clusters 1 and 4, while cluster 2 had significantly the highest BY under combined heat-drought stress. Cluster 2, representing moderately tolerant genotypes, recorded the highest values for PH (33 cm) and BY (295 g per plot) under the combined heat-drought stress.

For grain morphology and quality, our findings indicated no significant differences between clusters, except for K, where notable differences were observed under both stress conditions (Table 3). Under heat stress, the tolerant genotypes registered the highest concentration of K with 896 mg/kg; however, the maximum value of K under combined heat and drought stress was achieved by the moderately tolerant genotypes (998.90 mg/kg). Zn was significantly different between the clusters under heat stress conditions, with a maximum value of 48.31 mg/kg recorded by the moderately tolerant genotypes (cluster 2). Protein content was significantly different between the four clusters under combined heat-drought stress, with the highest value (22.58%) reached by the moderately susceptible genotypes (cluster 4).

## 3. Discussion

### 3.1. Agronomic Performance and Quality under Heat and Drought Stress

In the present study, a late planting strategy was used to synchronize the reproductive stage of chickpea plants with high temperatures. Drought stress was avoided by applying full irrigation. However, terminal heat-drought stress was induced by stopping irrigation at the start of the reproductive phase. The same methods have been widely applied in several studies in wheat [37], rice [38], lentil [33,35] and chickpea [31,32].

A significant decrease in chickpea yield was noticed under both stress conditions, which is in good alignment with previous studies in chickpea [39,40,41,42]. Indeed, the reduction of the harvest index can primarily be attributed to its positive correlation with the GY, as demonstrated in lentil [35] and canola [43]. Recently, similar findings were observed in lentil [33] with a decrease of 23.2% in grain yield due to the combined heat-drought stress and 37% in wheat because of the drought stress [44]. Plant height was positively correlated with biological yield under heat stress and severely affected by the combined heat-drought stress. Multistage phenotyping in chickpea demonstrated a negative effect of temperature rise (>30 °C) on this dynamic trait, which reaches its maximum during the first month of growth [45].

In our study, the first pod height and plant height were highly positively correlated; however, both parameters decreased simultaneously under heat and the combined heat-drought stresses. Similar results were identified by Müller et al. [46] showing the concentration of soybean plant fructification at the upper part due to the difficulty encountered by the intercepted photosynthesis active radiation to penetrate the vegetative canopy of a tall plant, thus increasing the height of the first pod insertion. This result demonstrated the negative effect of heat and combined heat-drought stresses on mechanical harvesting, given that these traits are required to improve the efficiency of this agricultural practice [47].

All the grain size parameters, including hundred-seed weight, experienced a slight decrease under stress treatments, but their high heritability under normal and stress conditions suggests their use in breeding programs of chickpea for more resilient varieties well adapted to heat and drought stress conditions. The same findings were reported in lentil, which showed a strong impact of genotypes on seed weight with high heritability (H^2^ = 0.93) [48,49].

The reduction in grain yield under stress is closely linked to a reduction in the number of pods and grains and grain size [50] induced by heat and/or water stress. The triggering effect of these stresses on this reduction started with the pod initiation stage [40]. It is manifested by a decrease either in the duration or pace of seed filling [51]. Cohen et al. [52] highlighted that the combination of drought and heat stress may shorten the life cycle of annual crops with premature seed production and reduced numbers and/or size, with less vegetative biomass, or by disrupting carbon assimilation and its transport in seeds. The biochemical mechanisms behind this morphological and yield decline are justified by the inhibition of cell division and carbohydrate synthesis in developing seeds via leaf sucrose supply downregulation, where the starch synthesizing enzyme was increased under stress conditions in chickpea [31,53]. Furthermore, Devasirvatham et al. [54] observed that abscisic acid downregulation due to heat stress may also decrease pollen viability, germination, flower retention, pod set, and therefore seed size and yield.

To cope with the devastating effects of water scarcity and rising temperatures, plants adopt different physiological mechanisms, such as decreased transpiration and modified root and shoot systems affecting micronutrient uptake and synthesis [34,55]. In the current study, our findings revealed a decrease in the protein content of chickpea seeds under both heat and combined heat-drought stress. Several studies have noted an improvement in nutritional quality via protein accumulation under water scarcity stress in chickpea [32,50,56], mung bean [57] and barley [58]. Heat stress also had a cumulative effect on up to 20% of soluble proteins in several food legumes, thus improving their nutritional quality [59], whereas other studies demonstrated a decrease in protein level [36,60,61,62]. These differences may be due to the duration and intensity of stress conditions and to the dry weight of the grains [53].

An increase in chickpea seed protein content, linked to total nitrogen, is more than a matter of stress concentration due to its significant negative correlation with grain yield and harvest index. It can be explained by the expression of drought and heat-induced proteins, including plant heat shock proteins (HSPs), having a protective role for non-native proteins from aggregation and strengthening the stability of the membrane, as well as the detoxification of reactive oxygen species (ROS), thus conferring tolerance to these abiotic stresses [63]. In the event of drought and/or heat stress, ROS induces HSP synthesis [64] and nitrogen uptake is enhanced [65]. In fact, 22 heat stress transcription factors (Hsfs) were discovered in chickpea [66]. A proteomic approach has allowed the identification of 147 differentially expressed proteins in chickpea likely involved in dehydration tolerance; in soybean and sorghum grains, a protein accumulation was recorded, especially in terms of HSP in thermotolerant genotypes [67,68,69].

Chickpea grain mineral profile: major changes in the present study were marked by a significant rise in the average concentrations of Na, Mn, and Mg for the heat and combined heat-drought stresses compared to the normal treatment. However, Fe, Zn, Se, K, Cu, and Ca changes were non-significant. Mg accumulation in chickpea grains under stress was not only the consequence of its negative correlation with biological yield and grain yield but also of its important role in chlorophyll synthesis and carbon metabolism [70]. The dynamism of this mineral is known for its very high phloem mobility towards growing sinks [71]. Indeed, the demand for Mg increases under high-light conditions, often accompanied by water and heat stress [72]. Magnesium deficiency in maize and wheat has demonstrated increased susceptibility to heat stress, which increased the shoot-to-root ratio, reduced soluble carbohydrates, and increased the activities of antioxidative enzymes against accumulated ROS [73].

Pretreatment with different sodium chloride concentrations alleviated drought resistance in alfalfa (*Medicago sativa* L.) by improving the water content of the aboveground soil and diminishing oxidative damage in the leaves under drought, which maintained the activity of PSII and PSI [74]. This finding can explain the accumulation of sodium in chickpea grains as a result of increased uptake and transport in the whole plant, favored by its great phloem mobility [75]. Dias et al. [76] also reported that after anthesis and during grain filling, the total Na content increased significantly under heat stress in durum wheat shoots. The positive correlation between Na content, grain size parameters, and hundred-seed weight under normal conditions revealed their close relationship, indicating seed number as an important criterion for the genetic selection of chickpea under stress conditions [77]. Similar findings were observed for foliar Mn content in wheat, canola, and soybeans as a response to heat stress [78]. In fact, during grain formation, a direct Mn xylem-ear transfer occurred [79]. Mn uptake remediates drought and harsh temperature impacts in numerous crops, as various enzymes such as Mn superoxide dismutase (Anti-ROS Production) and allantoate amidohydrolase (Anti-urea derivatives) are Mn-dependent. It enhances the photosynthesis process, improves nitrogen fixation, and maintains cell integrity by reducing malondialdehyde (MDA) content [80].

For other minerals, similar results were observed for durum wheat grains subjected to drought stress, where Zn and Cu remained unchanged [81]. Zn, Fe, Cu, K, Mn, and Ca were not correlated with grain yield in all treatments. This opens the possibility of breeding genotypes with high grain yields and high content of these minerals simultaneously. Zn and Fe may be biofortified concurrently due to the positive correlation observed in this study under normal conditions, which is consistent with the findings of several studies [32,33].

Our study also showed that iron decreased due to heat stress, which supports the results of an earlier study on chickpeas [32]. The impact of heat stress was also reductive in lentils [33,34] and more pronounced in combination with drought stress; however, our study revealed that the combined heat-drought stress had no significant effect on iron content when compared to heat stress treatment and normal conditions. Under heat and combined heat-drought stress, selenium had a negative correlation with biological and grain yields without any significant accumulation. This could be explained by its accentuated use in the event of these harsh conditions mobilizing its absorption. Selenium is widely involved in alleviating the adverse effects of these stresses in several species through the activation of the antioxidant defense system and the biochemical pathways of salicylic and jasmonic acid, its contribution to the formation of selenoproteins and glutathione peroxidase [82] and increasing the water uptake of the root system [71] at par with Na under combined stress [83]. Under these conditions, selenium also acted positively on improving protein levels, grain weight, and grain size [84,85].

Induced by abiotic post-anthesis stress, nutrient cycling varies among crop species, modifying several physiological processes in plants. Mineral patterns in grains are very complex and nutrient-specific, and the hypothesis of carbon reduction expressed by harvest index, grain yield reduction, and mineral accumulation is not true at 100%, as is the case for our findings regarding Mg, Mn, and Na accumulation, iron reduction, and stability for the other studied minerals. Their concentrations in plants may be attributed to the combination of their specific roles, phloem mobility, and post-anthesis uptake depending on the roots’ mass, growth, and conductance changes [65,81,86].

### 3.2. Stress Tolerance

PCA and hierarchical clustering have allowed the grouping of chickpea genotypes based on several stress indices. High GMP, MP, HARM, and STI were determinants for the selection of tolerant genotypes that had high potential yield under stress and non-stress conditions, given their positive correlation with Ys. PCA’s first dimension correlated positively with these indices and could be associated with yield performance and tolerance level under heat and combined heat-drought stress conditions. Genotypes with high PC1 values will have the ability to give high grain yields under normal as well as stress conditions. SSI is useful for the selection of sensitive genotypes to stress through a great loss in grain yield due to its negative correlation with Ys. In the present study, SSI was positively correlated (≤0.001) with PC2 under the two abiotic stresses. Consequently, the second dimension would differentiate genotypes according to their level of stress tolerance; thus, genotypes with a high PC2 are adequate for normal conditions (Table S6).

These stress indices have been widely used and found effective by combining them with other physiological and phenotypic parameters in many studies aiming at the genetic selection of water stress tolerance, especially in chickpea [41,87], lentil [35,88] and wheat [89,90]. High grain yield under stress conditions (Ys) was not sufficient to confirm the tolerance of genotypes by having a high yield stability index (YSI) and therefore very low TOL and SSI given their negative correlation, but rather to identify genotypes with good agronomic performance under stress worsening under normal conditions, as is the case for FLIP09-136C and FLIP09-227C under combined stress.

Genetic analysis of the clusters under combined heat-drought stress identified genotypes that maintained their tolerance level by shifting from heat to combined stress, in particular FLIP04-5, FLIP09-229C, FLIP09-222C, FLIP09-221C, FLIP09-274C, Mubarak, ILC12004, FLIP07-225C, ILC482, FLIP09-314C, and FLIP07-75 for tolerant genotypes, FLIP09-136C for moderately tolerant, Douyet for moderately susceptible, and FLIP07-187C, FLIP09-139C, FLIP09-197C, FLIP90-96C, FLIP07-218C, FLIP07-227C, and FLIP09-102C for susceptible. Other genotypes improved their tolerance with stable grain yield when exposed to combined heat-drought stress, in particular FLIP09-301C, ILC3397, FLIP09-81C, Zahour, ILC1302, FLIP09-96C, FLIP97-7, FLIP07-211C, and ILC263 upgrading from susceptible to moderately tolerant genotypes, FLIP09-111C and FLIP09-148C from sensitive to tolerant genotypes, FLIP09-227C changing from moderately susceptible to moderately tolerant, ILC533, Farihane, FLIP09-135C, Arifi, FLIP08-82C, FLIP09-290C, and FLIP07-221C change from susceptible to moderately tolerant. Other genotypes decreased their performance with the advent of water stress, including FLIP07-184C, FLIP09-304C, FLIP08-84C, and FLIP07-209C, from moderately tolerant to moderately susceptible genotypes. Substantially, a great part of the studied collection was resilient and has kept or improved its tolerance level under drought stress conditions; this implies that it could be a potential source of genes for resistance to heat and drought stresses. A recent study using genome-wide association has identified tolerance candidate genes in chickpea, allowing the selection of genotypes escaping water and heat stresses [91]. Indeed, drought induces an intensive expression of stress genes in tolerant genotypes, revealing a genetic predisposition to cope with it [25].

The comparison of the cluster’s mean mineral content under normal and stress environments distinguished potassium by its increased content concordantly with tolerance level. Potassium accumulation during grain filling was also detected in durum wheat under heat stress [76]. The present study revealed high genotypic and genotype-treatment influences for potassium, suggesting that the genes highly induced by environment are closely linked to the accumulation of this element in tolerant genotypes, explaining heritability decreases by moving from normal to stress conditions. Drought stress limited potassium acquisition in plants, which is crucial for their nutritional status. Zare et al. [92] showed that the application of potassium in corn significantly increased seed yield, hundred-seed weight, and grain number under drought stress conditions. Potassium is also involved in the molecular mechanisms of drought tolerance [93,94,95,96]. In the study of Azeem et al. [97], thirty-six chickpea genes were discovered in the encoding of the K transport system (channels and transporters) and the modulation of tolerant chickpea genotypes’ responses under abiotic stress. This genetic mechanism of stress endurance via potassium assimilation is realized by modulating water uptake. In addition, several studies demonstrated a regulation at the transcriptional level of the K system, in which assimilation correlates positively with water absorption [93,94,95,96]. These results suggest that there is a high genetic potential in the studied germplasm, enabling it to enhance its nutritional quality despite sinking yields under heat or combined stresses. The tolerant genotypes had higher potassium absorption, which reflected positively on the mobilization of water inside the roots and indirectly on grain yield.

## 4. Material and Methods

### 4.1. Plant Material and Field Description

Forty-three chickpea genotypes from an international collection available in the ICARDA GenBank were evaluated at the ICARDA experimental station in Marchouch, Morocco, for two crop seasons in 2015–16 and 2016–17. Details of the selected genotypes are presented in Table S9. The experimental site falls under the favorable rainfed agro-ecological zone (33°34′3.1″ N, 6°38′0.1″ W) and is characterized by a Mediterranean/warm temperate climate, abundant precipitation with an average rainfall of 450 mm, and temperatures varying between 7 and 32 °C. The soil is a clay vertisol type with a neutral pH ranging from 6.1 to 7.5 and an organic matter content ranging from 1.2 to 2.2%. A basal fertilizer (6% N, 30% P, and 20% K) dose of 80 kg/ha in the winter crop and 40 kg/ha in the spring crop and the recommended agronomic practices, including pest and weed management, were followed throughout the study period.

### 4.2. Experimental Design and Climate Data

**Figure 4:**
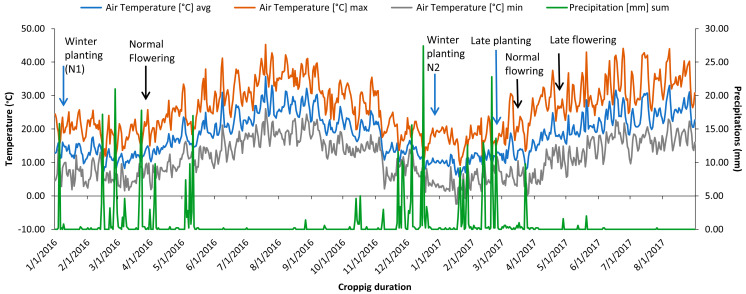
Max/min temperature and precipitation recorded during the study period at Marchouch, Morocco.

The study was carried out over two years. During the first year, only the normal planted experiment (N1) was taken up and sown in mid-January 2016 (Figure 4) in an alpha lattice design with two replications. Each genotype was planted in a plot consisting of two rows of 1.5 m length spaced at a 30 cm distance.

The same set was assessed during 2016/2017 in three independent environments. Normal planting (N2), late planting with irrigation (SI), and late planting without irrigation (SNI) Normal sown crops (N2) represented optimal growing conditions without any heat or water stress to the plants, whereas SI and SNI experiments exposed chickpea genotypes to heat stress and combined heat-drought stress, respectively. Sowing of the N2 experiment was taken up on 28 2016, while SI and SNI experiments were planted on 2 March 2017 to simulate water and heat stress at the reproductive stage. In both late planting experiments, the plants were synchronized with temperatures above 32 °C. Irrigation was performed on a regular basis to maintain water supply at field capacity using a sprinkler system throughout the crop duration in the experiment in which SI was exposed to high-temperature stress. In contrast, irrigation was stopped from the flower initiation phase onward in experiment SNI to impose water stress (<5 mm of rainfall during the reproductive stage) combined with temperature stress.

Assessment of the year’s effect in normal conditions was realized by studying two winter environments (N1 and N2). Average precipitation of 118 mm (2015/2016) and 139 mm (2016/2017) was well distributed during the reproduction phase, and temperatures remained cool below 20 °C during both seasons (Figure 4). The reproduction phase of late-planted chickpea genotypes started at the end of April and coincided with the start of a drought period that lasted until the harvest with temperatures rising to 32 °C, inducing two forms of stress: heat stress (SI) and combined heat-drought (SNI).

### 4.3. Data Collection

Observations were recorded on plant height (PH), first pod height (FPH), 100-seed weight (HSW), biological yield (BY), and seed yield (GY) on five plants selected randomly from each plot at maturity. Harvested grains were scanned to record grain morphology traits such as area, perimeter, length, and width using a scanner, CanoscanLide 220 (Canon, Paris, France), equipped with CSIRO grainscan software [98].

Stress tolerance of late-planted genotypes under heat stress (SI) and combined heat-drought stress (SNI) was assessed by the determination of several indices calculated based on seed yield under stress (YS) and normal (Yp) conditions of the same year. These indices, namely stress tolerance index (STI), geometric mean productivity (GMP), mean productivity (MP), tolerance index (TOL), harmonic mean (HARM), stress susceptibility index (SSI), and yield stability index (YSI), were calculated as per the below equations [99,100,101,102,103]:

Stress tolerance index (STI) = (Ypi × Ysi)/Yp^2^

Tolerance index (TOL) = Ypi − Ysi

Geometric mean productivity (GMP) = √Ypi × Ysi

Mean productivity (MP) = (Ypi + Ysi)/2

Harmonic mean (HARM) = 2(Yp × Ys)/Yp + Ys

Stress susceptibility index (SSI) = 1 − (Ys/Yp)/SI

Yield Stability Index (YSI) = Ys/Yp

where Ysi = yield of i genotype under stress conditions, Ypi = yield of i genotype under normal conditions, Ys = overall genotypic mean under stress conditions, and Yp = overall genotypic mean under normal conditions.

### 4.4. Protein Content Estimation

Protein content was determined using the Kjeldahl method [104]. 1 g of powdred chickpea was mineralized with concentrated sulfuric acid, the extract was alkalinized by 40% sodium hydroxide, and then the distilled ammonia was absorbed in a 4% boric acid solution and titrated by hydrochloric acid (0.1 N). The nitrogen amount was converted to total protein by multiplying by a factor of 6.25.

### 4.5. Mineral Estimation

Mineral composition was determined using a modified HNO_3_-H_2_O_2_ method [105,106]. The chickpea seeds were finely milled (0.2 mm) using a grinder (IKA, Staufen, Germany), and 0.5 g was digested with 0.7 mL of 70% nitric acid, digested at 90 °C for one hour, and added with 3 mL of hydrogen peroxide (H_2_O_2_) at 30% (*v*/*v*). Digestion extracts were filtered and diluted (1:10) with HCl 6M. Standard mix calibration concentrations were prepared. The mineral concentration was measured by inductively coupled plasma-optical emission spectroscopy (ICP-OES) using the ICP-7000 Duo (Thermo Fisher Scientific, Waltham, MA, USA).

### 4.6. Statistical Analysis

An analysis of variance (ANOVA) was performed using the general linear model (GLM) using IBM SPSS Statistics 23. Treatment means were compared by least significant difference (LSD) at *p* < 0.05. Pearson’s correlation coefficient was calculated by multivariate analysis for heat and combined heat-drought stress conditions. Heritability was calculated using MetaR (Multi-Environment Trial Analysis with R for Windows version 6.0) [107]. Hierarchical clustering using Ward’s squared Euclidean distance method was performed with the *dendextend* R package [108]. Principal component analysis (PCA) was performed using *Factoextra* [109] and *FactoMineR* [110] packages in R version 4.1.0 and RStudio version 1. 3.1093.

## 5. Conclusions

Heat and combined heat-drought stress significantly impacted the growth and productivity of chickpeas by decreasing plant height, biomass, and grain yield. Nevertheless, the nutritional quality was improved by accumulating proteins and numerous micronutrients such as Mg, Na, and Mn. Protein content increased under both stresses, increasing from 20.26% under normal conditions to 22.19% under heat stress and 21.94% under combined heat-drought stress, encouraging in-depth studies exploring the proteomic profile of chickpea.

Our findings revealed that only genotype and genotype-environment interaction factors had a significant impact on K content. Hundred-seed weight and grain size had high heritabilities under normal and stress conditions, suggesting them as candidate traits for selection criteria. Principal component analysis generated four clusters with different tolerance levels for the two stresses. FLIP04-5, FLIP09-222C, ILC12004, Moubarak, and ILC482 were identified together in the tolerant cluster under heat and combined heat-drought stress. The tolerance of a large part of the studied collection, i.e., FLIP09-81C, ILC3397, Zahour, FLIP97-7, and ILC263, under combined heat-drought stress was enhanced, demonstrating a genetic resistance predisposition. An accumulation of K content in the tolerant cluster of each stress is probably linked to one of the resistance scenarios. This element serves as a water mobilizer in the plant, helping it cope with these abiotic stresses. Further studies on resistance genes and their activation in chickpea deserve to be investigated, including the expression of K-encoding transport, which has a rather high heritability under normal conditions but is strongly affected under drought and heat stresses. Other chickpea nutritional parameters (amino and fatty acid profiles), technological (cooking time), and functional qualities (protein quality, starch quality) must be explored under drought and heat stress in future research.

## Figures and Tables

**Table 1:** Pearson’s correlation coefficient among yield stressed (Ys), yield optimal (Yp), and stress indices under heat stress (SI) (above) and combined heat-drought stress (SNI) (below) conducted during 2016–2017 at Marchouch.

	Ys	Yp	STI	TOL	GMP	MP	HARM	SSI	YSI
**Ys**	1	0.60 **	0.94 **	0.09	0.94 **	0.81 **	0.99 **	−0.74 **	0.74 **
**Yp**	0.58 **	1	0.80 **	0.85 **	0.83 **	0.95 **	0.71 **	0.06	−0.06
**STI**	0.91 **	0.83 **	1	0.38 *	0.99 **	0.94 **	0.98 **	−0.50 **	0.50 **
**TOL**	−0.04	0.79 **	0.33 *	1	0.42 **	0.65 **	0.23	0.55 **	−0.56 **
**GMP**	0.92 **	0.84 **	0.98 **	0.34 *	1	0.96 **	0.98 **	−0.48 **	0.48 **
**MP**	0.82 **	0.94 **	0.96 **	0.54 **	0.97 **	1	0.89 **	−0.24	0.24
**HARM**	0.97 **	0.74 **	0.97 **	0.18	0.99 **	0.92 **	1	−0.63 **	0.63 **
**SSI**	−0.82 **	−0.04	−0.53 **	0.57 **	−0.55 **	−0.37 *	−0.67 **	1	−1.00 **
**YSI**	0.82 **	0.04	0.53 **	−0.57 **	0.55 **	0.37 *	0.67 **	−1.00 **	1

** and * indicate significance at 0.01 and 0.05 levels; Ys, Yield under stress; Yp, yield under optimal conditions; STI, Stress tolerance index; TOL, Tolerance index; GMP, Geometric mean production; MP, Mean production; HARM, Harmonic mean; SSI, Stress tolerance index; YSI, Yield stress index.

**Table 2:** Eigenvalues and eigenvectors of the three PCA dimensions under heat and combined heat-drought stresses.

Traits	Heat Stress			Heat-Drought Stress		
	Dim.1	Dim.2	Dim.3	Dim.1	Dim.2	Dim.3
**Ys**	15.20	2.32	4.72	14.91	2.48	0.41
**Yp**	10.07	13.53	7.75	9.49	15.03	8.83
**STI**	15.98	0.09	15.92	15.32	0.63	29.93
**TOL**	1.95	30.87	23.71	0.80	35.15	16.98
**GMP**	16.18	0.28	1.62	15.71	0.56	1.46
**MP**	14.33	4.35	1.42	14.21	4.10	3.21
**HARM**	16.05	0.40	6.71	15.81	0.05	8.37
**SSI**	5.14	24.01	19.17	6.87	21.00	15.41
**YSI**	5.09	24.13	18.98	6.87	21.00	15.41
**Eigenvalue**	6.11	2.79	0.07	6.25	2.67	0.06
**Variance explained (%)**	67.95	31.09	0.75	69.39	29.68	0.68
**Total variance (%)**	67.95	99.04	99.80	69.39	99.07	99.75

Ys, Yield under stress; Yp, yield under optimal conditions; STI, Stress tolerance index; TOL, Tolerance index; GMP, Geometric mean production; MP, Mean production; HARM, Harmonic mean; SSI, Stress tolerance index; YSI, Yield stress index.

**Table 3:** Mean values of identified clusters for all parameters under heat and combined heat-drought stresses.

	Clusters	FPH	PH	BY	GY	HI	HSW	Area	Perimeter	Length	Width
		cm	cm	g/plot	g/plot	%	g	mm^2^	mm	mm	mm
**combined stress**	Cluster 1	17.19 a	28.24 b	236.08 ab	80.95 b	35.48 a	34.00 a	53.91 a	34.22 a	9.50 a	7.69 a
	Cluster 2	17.33 a	33.00 a	295.00 a	112.33 a	38.26 a	36.17 a	54.89 a	35.10 a	9.73 a	7.83 a
	Cluster 3	19.56 a	31.17 a	191.67 b	25.33 d	12.80 b	34.31 a	53.80 a	34.43 a	9.70 a	7.51 a
	Cluster 4	20.00 a	29.94 ab	219.44 ab	55.17 c	26.55 a	30.73 a	49.78 a	32.78 a	9.07 a	7.43 a
**heat stress**	Cluster 1	17.10 a	28.65 a	238.75 a	89.10 a	37.56 a	31.09 a	49.94 a	33.10 a	9.19 a	7.44 a
	Cluster 2	16.30 a	28.85 a	216.00 a	63.70 b	30.36 b	32.68 a	51.53 a	33.35 a	9.25 a	7.54 a
	Cluster 3	16.36 a	28.09 a	223.86 a	63.43 b	28.60 b	36.56 a	56.41 a	35.10 a	9.75 a	7.85 a
	Cluster 4	16.64 a	28.43 a	221.82 a	46.09 c	21.55 c	32.20 a	50.88 a	33.08 a	9.18 a	7.47 a
	**Clusters**	**Protein**		**Fe**	**Zn**	**Se**	**K**	**Cu**	**Mn**	**Ca**	**Na**
		**%**		**mg/kg**	**mg/kg**	**mg/kg**	**mg/kg**	**mg/kg**	**mg/kg**	**mg/kg**	**mg/kg**
**combined stress**	Cluster 1	21.61 ab		55.37 a	37.88 a	0.14 a	854.87 ab	4.10 a	26.47 a	1021.98 a	273.10 a
	Cluster 2	22.04 a		65.59 a	43.17 a	0.10 a	998.90 a	5.30 a	29.93 a	1130.51 a	257.42 a
	Cluster 3	20.26 b		56.14 a	40.05 a	0.16 a	689.44 b	3.14 a	28.72 a	882.01 a	276.46 a
	Cluster 4	22.58 a		53.06 a	48.03 a	0.13 a	865.00 ab	4.04 a	29.92 a	1098.57 a	291.82 a
**heat stress**	Cluster 1	21.73 a		48.96 a	45.24 ab	0.14 a	896.27 a	4.09 a	28.80 a	1086.87 a	266.76 a
	Cluster 2	22.01 a		52.24 a	48.31 a	0.15 a	845.35 ab	3.78 a	27.13 a	1223.92 a	257.71 a
	Cluster 3	22.23 a		50.10 a	39.31 ab	0.14 a	784.75 ab	3.99 a	28.29 a	1098.39 a	273.16 a
	Cluster 4	22.74 a		59.55 a	36.24 b	0.15 a	748.08 b	3.95 a	30.69 a	1051.36 a	298.45 a

Letters “a” and “b” indicate significant differences at 0.05 probability level using Duncan test; FPH, First pod height; PH, Plant Height; BY, Biological yield; GY, Grain yield; HI, Harvest index; HSW, Hundred seed weight.

## Data Availability

Datasets generated and/or analyzed during the current study are available from the corresponding author upon reasonable request.

## Supplementary Materials

The following supporting information can be downloaded at: https://www.mdpi.com/article/10.3390/plants12213726/s1, Table S1. Combined analysis of variance (expressed as percentage) for different traits among 43 chickpea accessions at across normal and heat stress and combined heat–drought stress experiments conducted at Marchouch during 2016–2017; Table S2. Analysis of variance (expressed as percentage) for measured traits across normal conditions of the two years 2015–2016 and 2016–2017; Table S3. Range, mean ± SE and heritability for different traits among 43 chickpea accessions in the four environments planted at Marchouch; Table S4. Correlation coefficient between yield, grain caliber and quality parameters in chickpea under heat stress trial (above diameter) and combined heat–drought stress (below) trial at Marchouch during 2016–2017; Table S5. Correlation coefficient between yield, grain caliber and quality parameters in chickpea under N2 (2016–2017) (above diameter) and N1 (2015–2016) (below) at Marchouch; Table S7. Yield under stress, yield potential and stress indices of 43 accessions of chickpea under Heat stress grown at Marchouch during 2016–2017; Table S8. Yield under stress, yield potential and stress indices of 43 accessions of chickpea under combined heat–drought stress grown at Marchouch during the season 2016–2017; Table S9: Chickpea genotypes identification.
