# Supplementary material for: Impact of Terminal Heat and Combined Heat-Drought Stress on Plant Growth, Yield, Grain Size, and Nutritional Quality in Chickpea (Cicer arietinum L.)
Source: Plants (Basel). 2023 Oct 30;12(21):3726. doi: 10.3390/plants12213726 (PMC10650860; doi:10.3390/plants12213726)
Supplement: Supplementary file 1 [file plants-12-03726-s001.zip › plants-2617320-supplementary.pdf]

Supplementary Material

Table S1. Combined analysis of variance (expressed as percentage) for different traits among 43 chickpea accessions at across normal and heat stress and combined heat-drought stress experiments conducted at Marchouch during 2016–2017

	Df	FPH	PH	BY	GY	HI	HSW	Area	Perimeter	Length	Width	Protein
**Treatments**	2	78.42***	85.54***	72.95***	59.38***	2.34**	4.44***	1.82***	2.08***	0.95**	4.98***	25.61***
**Genotype**	42	10.72***	6.62***	10.64**	15.66**	54.88***	74.49***	79.14***	78.79***	79.48***	75.65***	27.95**
**G*T**	74	5.35ns	4.71**	7.36ns	9.95ns	20.78ns	15.02***	14.17***	14.13***	14.24***	14.75***	21.54ns
**LSD**		3.08	3.34	24.29	32.99	8.33	5.54	6.92	2.17	0.60	0.49	5.61

Table S1. Continued

	Df	Fe	Zn	Se	K	Cu	Mn	Ca	Mg	Na
**Treatments**	2	1.96ns	0.67ns	1.09ns	1.57ns	0.12ns	5.25**	1.38ns	5.72**	6.40**
**Genotype**	42	19.10ns	17.83ns	25.96*	22.31*	17.70ns	22.84ns	17.32ns	14.45ns	14.93ns
**G*T**	74	37.36ns	45.92*	39.98ns	46.79**	34.60ns	33.48ns	39.04ns	41.47ns	36.93ns
**LSD**		25.49	6.80	0.03	18.29	1.24	12.39	490.00	370.39	150.70

*, **, ns indicate significance at 0.01 and 0.001 probability levels, and non-significant, respectively; PH, Plant height; FPH, First pod height; BY, Biological yield; SY, Seed yield; HI, Harvest index.

Table S2. Analysis of variance (expressed as percentage) for measured traits across normal conditions of the two years 2015-2016 and 2016-2017.

	Df	FPH	PH	BY	GY	HI	HSW	Area	Perimeter	Length	Width	Protein
**Year**	1	26.00**	0.74ns	35.23**	0.19ns	52.77**	5.80**	6.77**	7.41**	27.73**	11.97**	32.89**
**Gen**	42	44.20**	57.61**	33.46**	45.32*	15.61*	87.29**	88.02**	87.13**	67.50**	81.72**	28.19**
**Year*Gen**	41	9.57ns	17.38ns	10.10ns	16.86ns	14.43ns	5.33**	3.78**	4.23**	3.73**	4.69**	16.81ns
**LSD**		11.15	6.42	23.46	30.94	18.84	3.94	2.67	1.34	1.39	1.43	5.23

Table S2. Continued

	Df	Fe	Zn	Se	K	Cu	Mn	Ca	Mg	Na
**Year**	1	6.21*	9.37**	0.96ns	1.45ns	52.05**	22.62**	9.68**	1.21ns	26.22**
**Gen**	42	23.61ns	30.33ns	26.98ns	35.08**	15.44ns	22.22ns	21.72ns	28.17*	20.98ns
**Year*Gen**	41	22.24ns	22.78ns	36.28*	28.58*	9.10ns	24.56ns	27.54ns	27.52*	22.16ns

**LSD**	15.16	14.82	34.10	16.95	2.78	9.70	340.00	290.70	29.70

*, **, ns indicate significance at 0.01 and 0.001 probability levels, and non-significant, respectively; PH, Plant height; FPH, First pod height; BY, Biological yield; SY, Seed yield; HI, Harvest index.

Table S3. Range, mean±SE and heritability for different traits among 43 chickpea accessions in the four environments planted at Marchouch

Trait		Normal1	Normal2	Heat stress (SI)	Combined heat-drought stress (SNI)
**FPH(cm)**	Min	13.67	21	9.5	9.5
	Max	40.67	43	23.5	25.5
	Mean	26.73b±0.40	32.44a±0.41	16.64d±0.37	18.03c±0.4
	CV	14.23	9.28	19.37	13.49
	LSD	6.06	4.47	2.96	4.21
	H^2	0.6	0.54	0.21	0.72
**PH(cm)**	Min	40	40	19.5	17
	Max	60	60	37.5	36.5
	Mean	49.77a±0.44	49.17a±0.46	28.51b±0.37	29.12b±0.44
	CV	6.95	5.84	10.53	12.75
	LSD	5.41	4.91	3.69	4.5
	H^2	0.59	0.71	0.37	0.38
**BY(g/plot)**	Min	120	35	10	10
	Max	699	790	330	395
	Mean	329.61b±7.82	509.00a±13.37	227.03c±6.15	231.89c±7.82
	CV	29.67	21.74	25.5	31.93
	LSD	125.54	136.25	52.48	55.82
	H^2	0.39	0.38	0.24	0.17
**GY(g/plot)**	Min	21.8	38	10	9
	Max	356.9	340	116	142
	Mean	158.35a±3.34	165.55a±6.03	68.68b±3.01	72.15b±3.35
	CV	35.01	29.82	37.25	37.95
	LSD	59.62	61.97	32.89	36.66
	H^2	0.29	0.4	0.57	0.57
**HI(%)**	Min	4.69	6.91	7.83	5.45
	Max	76	64.02	47.14	55.24
	Mean	47.83a±1.36	32.63b±0.94	30.52b±1.19	31.87b±1.36
	CV	15.83	23.07	28.25	22.18
	LSD	5.18	9.8	12.84	12.29
	H^2	0.12	0.41	0.68	0.84
**HSW(g)**	Min	12.9	12.81	13.37	12
	Max	52.1	53.74	51.61	53
	Mean	33.43b±0.84	36.66a±0.77	33.39b±0.88	33.16b±0.84
	CV	3.93	3.87	9.95	9.86
	LSD	2.69	2.92	7.54	7.48
	H^2	0.98	0.98	0.88	0.87
**Area(mm^2)**	Min	29.48	29.13	28.89	30.24
	Max	73.02	75.4	74.42	73.83

	Mean	51.52c±0.96	55.26a±0.88	52.56b±1.02	52.73b±0.96
	CV	2.82	2.48	7.84	2.83
	LSD	3.06	2.83	9.3	3.06
	H^2	0.98	0.98	0.86	0.98
**Perimeter(mm)**	Min	25.36	25.77	24.82	25.46
	Max	40.71	41.3	40.48	40.33
	Mean	33.39c±0.32	34.77a±0.30	33.81b±0.34	33.85b±0.32
	CV	1.48	1.18	4.35	2.13
	LSD	1.04	0.84	3.31	1.64
	H^2	0.98	0.98	0.85	0.96
**Length(mm)**	Min	6.59	7.07	6.9	7.08
	Max	10.66	11.79	11.29	11.21
	Mean	8.72c±0.09	9.58a±0.08	9.39b±0.09	9.39b±0.09
	CV	1.51	1.27	4.51	1.92
	LSD	0.28	0.25	0.95	0.41
	H^2	0.98	0.98	0.83	0.97
**Width(mm)**	Min	5.8	5.6	5.73	5.68
	Max	8.75	8.93	8.96	8.96
	Mean	7.52c±0.07	7.91a±0.06	7.60b±0.07	7.61b±0.07
	CV	0.54	1.3	3.94	2.23
	LSD	0.24	0.21	0.67	0.38
	H^2	0.97	0.98	0.87	0.95
**Protein (g/100gDM)**	Min	20.41	17.01	18.61	18.19
	Max	26	24.67	24.62	24.06
	Mean	22.19a±0.14	20.22b±0.17	22.13a±0.13	21.85a±0.14
	CV	3.09	6.89	5.25	4.69
	LSD	1.18	1.65	0.73	1.44
	H^2	0.69	0.34	0.1	0.47
**Fe(mg/kg)**	Min	34.02	27.2	24.33	27.43
	Max	103.18	87.39	83.55	83.57
	Mean	50.32c±1.43	57.35a±1.50	51.75bc±1.74	55.23ab±1.44
	CV	22.08	24.39	26.89	22.23
	LSD	52.81	30.1	16.81	11.07
	H^2	0.1	0.1	0.33	0.2
**Zn(mg/kg)**	Min	15.37	13.72	13.92	15.01
	Max	75.13	67.75	68.7	65.77
	Mean	31.93b±1.33	38.78a±1.56	42.13a±1.54	41.24a±1.33
	CV	26.85	37.02	32.89	27.57
	LSD	18.59	6.55	32	10.78
	H^2	0.1	0.1	0.1	0.22
**Se(mg/kg)**	Min	0.01	0.06	0.04	0.05
	Max	0.28	0.31	0.32	0.31
	Mean	0.15a±0.01	0.14ab±0.01	0.14ab±0.01	0.13b±0.01

	CV	30.1	38.66	36.23	32.94
	LSD	0.06	0.05	0.05	0.07
	H^2	0.46	0.22	0.29	0.53
**K(mg/kg)**	Min	446.09	433.06	431.2	430.28
	Max	1329.3	1514.02	1186.1	1409.56
	Mean	926.63a±21.50	874.61ab±22.74	823.99b±18.72	856.81b±21.51
	CV	12.93	19.94	21.3	21.71
	LSD	211.33	239.89	358.65	165.3
	H^2	0.73	0.45	0.1	0.19
**Cu(mg/kg)**	Min	3.38	1.18	1.1	0.98
	Max	11.39	11.3	7.36	10.83
	Mean	8.67a±0.24	4.13b±0.26	3.99b±0.18	4.09b±0.24
	CV	16.47	58.36	39.03	53.65
	LSD	1.46	5.03	1.07	1.41
	H^2	0.26	0.1	0.11	0.11
**Mn(mg/kg)**	Min	21.46	7.11	7.55	7.7
	Max	43.81	44.03	39.89	38.67
	Mean	33.95a±0.76	24.77c±0.95	28.69b±0.69	27.77b±0.76
	CV	19.44	29.46	21.86	25.06
	LSD	14.1	9.95	13.6	14.44
	H^2	0.1	0.44	0.1	0.1
**Ca(mg/kg)**	Min	665.27	639.93	695.25	674.54
	Max	1701.59	1659.07	1648.67	1682.1
	Mean	1317.41a±31.04	1108.30b±24.13	1106.57b±29.26	1043.03b±31.05
	CV	18.68	20.17	22.43	25.87
	LSD	300.29	506.17	293.07	661.4
	H^2	0.36	0.1	0.37	0.1
**Mg(mg/kg)**	Min	399.04	809.63	893.58	879.18
	Max	1855.37	1666.34	1727.23	1677.8
	Mean	1290.84ab±24.20	1206.11b±23.07	1323.85a±21.69	1247.79ab±24.2
	CV	16.58	17.71	12.47	16.29
	LSD	342.71	452.03	236.23	187.08
	H^2	0.61	0.1	0.51	0.2
**Na(mg/kg)**	Min	94.66	88.27	89.6	89.71
	Max	466.01	434.15	418.74	434.60
	Mean	334.67a±8.89	230.07c±9.33	273.09b±9.25	278.02b±8.89
	CV	26.19	34.79	30.37	28.74
	LSD	40.7	78.48	182.42	183.21
	H^2	0.1	0.24	0.1	0.1

The letters “a” and “b” indicate significant differences using Duncan test at 0.05 probability level; PH, Plant heigh ; FPH, First pod height ; BY, Biological yield ; SY, Seed yield; HI: Harvest index.

Table S4. Correlation coefficient between yield, grain caliber and quality parameters in chickpea under heat stress trial (above diameter) and combined heat-drought stress (below) trial at Marchouch during 2016-2017.

	FPH	PH	BY	GY	HI	HSW	Area	Perimeter	Length	Width	Protein	Fe	Zn	Se	K	Cu	Mn	Ca	Mg	Na
**FPH**	1	.59**	.24*	-.001	-.16	.19	.16	.20	.17	.22	.04	.08	-.11	-.12	.04	.04	-.11	.09	-.05	.03
**PH**	.49**	1	.10	.06	.03	.24*	.24	.25*	.23	.25*	-.05	-.12	-.17	.03	.13	.14	-.05	.15	.09	-.002
**BY**	.17	.37**	1	.56**	.24*	.03	.02	.06	.01	.11	-.004	.01	-.12	.04	-.02	.10	.04	.14	.09	-.06
**GY**	-.29**	.04	.56**	1	.88**	-.15	-.16	-.13	-.14	-.08	-.22*	-.21	.05	.12	.09	-.03	-.03	.10	.09	-.21
**HI**	-.48**	-.16	.08	.79**	1	-.25	-.26*	-.26*	-.25*	-.24	-.24*	-.27*	.10	.15	.15	-.07	-.06	.08	.07	-.22*
**HSW**	.07	.13	.17	.10	-.09	1	.99**	.98**	.96**	.98**	-.10	-.15	-.14	-.02	-.13	-.11	-.08	.10	-.07	-.14
**Area**	.11	.12	.17	.10	-.10	.94**	1	.99**	.98**	.97**	-.08	-.16	-.14	-.01	-.09	-.10	-.07	.11	-.06	-.14
**Perimeter**	.13	.15	.21	.11	-.13	.92**	.99**	1	.99**	.97**	-.06	-.17	-.12	-.05	-.06	-.11	-.06	.11	-.07	-.13
**Length**	.14	.13	.20	.08	-.16	.89**	.97**	.99**	1	.93**	-.05	-.16	-.11	-.04	-.04	-.11	-.07	.12	-.07	-.11
**Width**	.12	.15	.18	.13	-.08	.92**	.97**	.95**	.90**	1	-.10	-.17	-.15	-.04	-.10	-.11	-.06	.07	-.06	-.16
**Protein**	.26*	.17	.19	-.13	-.29**	-.07	-.15	-.16	-.20	-.07	1	-.10	.17	-.03	.10	.37**	.18	-.03	-.05	.02
**Fe**	-.18	.07	.22	.19	.07	.12	.01	.02	.04	-.03	.10	1	-.04	-.20	-.23*	-.14	-.15	-.25*	-.13	-.13
**Zn**	.23*	.10	-.05	-.13	-.14	-.03	-.02	-.04	-.04	-.02	.10	-.13	1	-.13	-.08	.06	-.01	.003	.19	.13
**Se**	-.07	-.06	-.40**	-.26*	.01	.29*	.29*	.24	.24	.26*	-.29**	-.00	-.02	1	.05	.01	.15	.21	.11	.13
**K**	-.01	.12	.03	.06	.08	-.07	-.08	-.08	-.13	-.01	.03	-.10	-.08	.15	1	.32**	.29**	.08	.00	.11
**Cu**	.14	-.02	.03	-.02	-.04	.08	.06	.05	.04	.08	.07	-.18	.05	-.10	.09	1	.40**	.03	-.08	.10
**Mn**	.10	-.04	.04	.00	-.09	.08	.11	.10	.10	.12	.08	-.04	.05	-.10	.19	.14	1	-.03	.17	.34**
**Ca**	.19	-.03	-.18	-.15	-.09	-.05	-.05	-.06	-.06	-.03	.08	.05	.05	.21	.30**	.04	.06	1	.47**	.04
**Mg**	.10	-.06	-.25*	-.23*	-.18	.09	.13	.12	.14	.10	.10	-.14	.04	.12	-.13	.09	.13	.33**	1	-.03
**Na**	.09	.08	-.12	-.06	.01	.10	.13	.12	.11	.14	-.14	-.05	.11	.24*	-.05	.20	.22	.13	.22	1

**. Correlation is significant at the 0.01 level (2-tailed).

*. Correlation is significant at the 0.05 level (2-tailed).

Table S5. Correlation coefficient between yield, grain caliber and quality parameters in chickpea under N2 (2016-2017) (above diameter) and N1 (2015-2016) (below) at Marchouch

	FPH	PH	BY	GY	HI	HSW	Area	Perimeter	Length	Width	Protein	Fe	Zn	Se	K	Cu	Mn	Ca	Mg	Na
**FPH**	1	**.74****	.06	**-.28****	**-.43****	.13	.09	.09	.03	.18	-.06	-.15	-.09	**.24***	**.26***	.01	-.10	.09	.02	.20
**PH**	**.50****	1	.10	**-.26***	**-.47****	**.22***	.20	.20	.14	**.29****	.15	-.13	-.13	**.23***	.15	.06	-.17	-.00	-.00	.04
**BY**	-.06	.09	1	**.66****	-.06	.05	-.01	-.00	-.04	.08	.01	.14	-.08	.05	-.01	-.05	.04	.07	-.08	.02
**GY**	-.07	.12	**.85****	1	**.69****	-.03	-.06	-.06	-.05	-.07	-.03	.14	-.02	.01	-.14	-.15	.08	.18	-.04	-.03
**HI**	-.02	.09	-.01	**.49****	1	-.13	-.11	-.11	-.05	**-.23***	-.06	.07	.04	-.01	-.14	-.17	.05	.15	.04	-.04
**HSW**	.19	-.06	-.05	-.03	-.00	1	**.99****	**.98****	**.95****	**.97****	.09	.04	-.02	.06	.14	.08	.02	-.06	.01	**-.28***
**Area**	.18	-.06	-.08	-.07	-.06	**.99****	1	**.99****	**.98****	**.96****	.09	.04	-.02	.05	.13	.10	.05	-.07	.01	**-.29****
**Perimeter**	.20	-.06	-.08	-.08	-.06	**.99****	**.99****	1	**.99****	**.96****	.08	.05	-.03	.05	.12	.09	.05	-.06	.01	**-.28***
**Length**	.17	-.08	-.10	-.10	-.09	**.96****	**.97****	**.98****	1	**.90****	.05	.04	-.02	.04	.10	.07	.08	-.06	-.01	**-.30****
**Width**	.19	-.02	-.03	-.02	-.01	**.97****	**.98****	**.97****	**.92****	1	.14	.05	-.06	.07	.15	.12	-.01	-.06	.03	**-.24***
**Protein**	-.05	-.09	**-.28***	**-.34****	-.21	.12	.15	.14	.19	.07	1	.12	.18	.11	-.05	.07	.16	-.11	.06	.08
**Fe**	-.03	.09	.00	.01	.02	-.19	-.19	-.19	-.15	-.2	.07	1	**.30****	-.01	-.12	.09	.06	-.04	.09	.09
**Zn**	.04	**.24***	-.11	-.17	-.10	.03	.06	.05	.05	.06	.11	**.40****	1	.09	-.01	-.12	**.24***	.00	.16	.19
**Se**	-.04	.05	.06	-.06	-.18	-.18	-.19	-.20	**-.22***	-.18	.18	-.13	-.20	1	.02	.11	-.17	.17	.00	.03
**K**	.19	.08	-.16	-.04	.18	**.32****	**.30****	**.29****	**.27***	**.32****	-.06	.02	-.07	-.03	1	.01	.03	.09	.00	.11
**Cu**	.14	.13	-.11	.02	.17	**.27***	**.25***	**.23***	.22	**.25***	.01	.05	-.02	-.05	**.73****	1	.04	-.16	-.15	-.19
**Mn**	.02	-.10	-.05	-.00	.07	.11	.11	.10	.08	.12	.09	.12	.02	-.03	**.38****	**.25***	1	-.16	.02	.13
**Ca**	.15	.12	.01	.14	**.22***	.07	.04	.03	.04	.07	**-.22***	.05	-.13	**-.26***	**.66****	.70**	.14	1	.18	.09
**Mg**	.19	.19	-.07	.10	**.26***	.22	.19	.18	.17	**.22***	-.17	.08	-.06	-.11	**.84****	**.86****	**.28****	**.86****	1	**.25***
**Na**	.10	.00	.15	.18	.07	-.02	-.04	-.03	-.02	-.04	-.01	.05	-.01	.02	.15	.06	.10	.06	.14	1

**. Correlation is significant at the 0.01 level (2-tailed).

*. Correlation is significant at the 0.05 level (2-tailed).

Table S6. Correlation traits PC1 and PC2 under Heat stress and Combined Heat-drought stress

	Heat stress		Combined Heat-drought stress	
	PC1	PC2	PC1	PC2
**GMP**	0.99**	-	0.99**	-
**HARM**	0.99**	-	0.99**	-
**STI**	0.99**	-	0.98**	-
**Ys**	0.96**	-	0.97**	-
**MP**	0.94**	0.35*	0.94**	0.33*
**Yp**	0.78**	0.62**	0.77**	0.63**
**YSI**	0.56**	-0.82**	0.66**	-0.75**
**TOL**	0.35*	0.93**	-	0.97**
**SSI**	-0.56**	0.82**	-0.66**	0.75**

***at 0.05, ** at 0.001**

Table S7. Yield under stress, yield potential and stress indices of 43 accessions of chickpea under Heat stress grown at Marchouch during 2016-2017.

Genotypes	Ys	Yp	STI	TOL	GMP	MP	HARM	SSI	YSI	Cluster
**FLIP04-5**	91	226	0.77	135	143.41	158.5	129.75	0.92	0.4	Cluster_1
**FLIP07-225C**	76	209.5	0.59	133.5	126.18	142.75	111.54	0.98	0.36	Cluster_1
**FLIP07-75**	72.5	168.5	0.46	96	110.53	120.5	101.38	0.88	0.43	Cluster_1
**FLIP09-221C**	95.5	196.5	0.7	101	136.99	146	128.53	0.79	0.49	Cluster_1
**FLIP09-222C**	108	177.5	0.71	69.5	138.46	142.75	134.29	0.6	0.61	Cluster_1
**FLIP09-229C**	98.5	208.5	0.77	110	143.31	153.5	133.79	0.81	0.47	Cluster_1
**FLIP09-274C**	83.5	220.5	0.69	137	135.69	152	121.13	0.96	0.38	Cluster_1
**FLIP09-314C**	90.5	157	0.53	66.5	119.2	123.75	114.82	0.65	0.58	Cluster_1
**ILC12004**	84	191	0.6	107	126.66	137.5	116.68	0.86	0.44	Cluster_1
**ILC482**	77.5	193	0.56	115.5	122.3	135.25	110.59	0.92	0.4	Cluster_1
**Moubarak**	90	202	0.68	112	134.83	146	124.52	0.85	0.45	Cluster_1
**FLIP07-209C**	56.5	139	0.29	82.5	88.62	97.75	80.34	0.92	0.41	Cluster_2
**FLIP08-84C**	63.5	139.5	0.33	76	94.12	101.5	87.27	0.84	0.46	Cluster_2
**FLIP09-136C**	64	137.5	0.33	73.5	93.81	100.75	87.34	0.82	0.47	Cluster_2
**FLIP09-227C**	72	126	0.34	54	95.25	99	91.64	0.66	0.57	Cluster_2
**ILC533**	62.5	125	0.29	62.5	88.39	93.75	83.33	0.77	0.5	Cluster_2
**Douyet**	59	167.5	0.37	108.5	99.41	113.25	87.26	1	0.35	Cluster_3
**FLIP07-184C**	56	165	0.34	109	96.12	110.5	83.62	1.02	0.34	Cluster_3
**FLIP07-211C**	56.5	174.5	0.37	118	99.29	115.5	85.36	1.04	0.32	Cluster_3
**FLIP09-301C**	71	194.5	0.52	123.5	117.51	132.75	104.03	0.98	0.37	Cluster_3
**FLIP09-304C**	54	165	0.33	111	94.39	109.5	81.37	1.04	0.33	Cluster_3
**FLIP09-81C**	67.5	194.5	0.49	127	114.58	131	100.22	1.01	0.35	Cluster_3
**FLIP09-96C**	59	204	0.45	145	109.71	131.5	91.53	1.1	0.29	Cluster_3
**FLIP97-7**	58.5	204.5	0.45	146	109.38	131.5	90.98	1.1	0.29	Cluster_3
**ILC1302**	44.5	276.5	0.46	232	110.92	160.5	76.66	1.29	0.16	Cluster_3
**ILC263**	60.5	190	0.43	129.5	107.21	125.25	91.78	1.05	0.32	Cluster_3

**ILC3397**	66.5	202.5	0.5	136	116.04	134.5	100.12	1.04	0.33	Cluster_3
**Zahour**	59.5	212.5	0.47	153	112.44	136	92.97	1.11	0.28	Cluster_3
**Arifi**	36	150	0.2	114	73.48	93	58.06	1.17	0.24	Cluster_4
**Farihane**	40.5	169	0.26	128.5	82.73	104.75	65.34	1.17	0.24	Cluster_4
**FLIP07-187C**	23	73.5	0.06	50.5	41.12	48.25	35.04	1.06	0.31	Cluster_4
**FLIP07-218C**	29	91.5	0.1	62.5	51.51	60.25	44.04	1.05	0.32	Cluster_4
**FLIP07-221C**	25.5	107.5	0.1	82	52.36	66.5	41.22	1.18	0.24	Cluster_4
**FLIP07-227C**	23.5	148.5	0.13	125	59.07	86	40.58	1.3	0.16	Cluster_4
**FLIP08-82C**	39	105.5	0.15	66.5	64.14	72.25	56.95	0.97	0.37	Cluster_4
**FLIP09-102C**	36.5	102.5	0.14	66	61.17	69.5	53.83	0.99	0.36	Cluster_4
**FLIP09-111C**	40	163.5	0.24	123.5	80.87	101.75	64.28	1.16	0.24	Cluster_4
**FLIP09-135C**	42	134.5	0.21	92.5	75.16	88.25	64.01	1.06	0.31	Cluster_4
**FLIP09-139C**	29	137.5	0.15	108.5	63.15	83.25	47.9	1.22	0.21	Cluster_4
**FLIP09-148C**	35	169	0.22	134	76.91	102	57.99	1.22	0.21	Cluster_4
**FLIP09-197C**	25	56	0.05	31	37.42	40.5	34.57	0.85	0.45	Cluster_4
**FLIP09-290C**	29.86	115.5	0.13	85.64	58.72	72.68	47.45	1.14	0.26	Cluster_4
**FLIP90-96**	22	149	0.12	127	57.25	85.5	38.34	1.31	0.15	Cluster_4

Ys, seed yield under heat stress; Yp, seed yield in normal condition; HI, Harvest index; GMP, geometric mean productivity; MP, mean productivity; TOL, tolerance index; STI, stress tolerance index; HARM, harmonic mean; SSI, Stress susceptibility index; YSI, Yield Stability Index. Cluster represents tolerance level of chickpea accessions (cluster1, tolerant genotypes; cluster2, moderately tolerant, cluster3, moderately susceptible, cluster4, susceptible genotypes).

Table S8. Yield under stress, yield potential and stress indices of 43 accessions of chickpea under combined heat-drought stress grown at Marchouch during the season 2016-2017.

Genotypes	Ys	Yp	STI	TOL	GMP	MP	HARM	SSI	YSI	Cluster
**FLIP09-274C**	50	220.5	0.41	170.5	105	135.25	81.52	1.25	0.23	Cluster_1
**FLIP04-5**	53	226	0.45	173	109.44	139.5	85.86	1.24	0.23	Cluster_1
**FLIP09-81C**	63.5	194.5	0.46	131	111.13	129	95.74	1.09	0.33	Cluster_1
**ILC3397**	71.5	202.5	0.54	131	120.33	137	105.68	1.04	0.35	Cluster_1
**Zahour**	72	212.5	0.57	140.5	123.69	142.25	107.56	1.07	0.34	Cluster_1
**FLIP09-314C**	73	157	0.43	84	107.06	115	99.66	0.86	0.46	Cluster_1
**ILC12004**	73.5	191	0.52	117.5	118.48	132.25	106.15	0.99	0.38	Cluster_1
**Moubarak**	73.5	202	0.55	128.5	121.85	137.75	107.78	1.03	0.36	Cluster_1
**ILC482**	76	193	0.55	117	121.11	134.5	109.06	0.98	0.39	Cluster_1
**FLIP97-7**	76.5	204.5	0.58	128	125.08	140.5	111.35	1.01	0.37	Cluster_1
**ILC263**	78.5	190	0.56	111.5	122.13	134.25	111.1	0.95	0.41	Cluster_1
**FLIP09-111C**	79	163.5	0.48	84.5	113.65	121.25	106.53	0.83	0.48	Cluster_1
**FLIP07-211C**	80.5	174.5	0.52	94	118.52	127.5	110.17	0.87	0.46	Cluster_1
**FLIP09-222C**	81	177.5	0.54	96.5	119.91	129.25	111.24	0.88	0.46	Cluster_1
**FLIP09-148C**	81.5	169	0.51	87.5	117.36	125.25	109.97	0.84	0.48	Cluster_1
**FLIP09-301C**	83.5	194.5	0.61	111	127.44	139	116.84	0.92	0.43	Cluster_1
**FLIP09-229C**	84.5	208.5	0.66	124	132.73	146.5	120.26	0.96	0.41	Cluster_1
**FLIP09-221C**	87	196.5	0.64	109.5	130.75	141.75	120.6	0.9	0.44	Cluster_1
**FLIP07-75**	91.5	168.5	0.58	77	124.17	130	118.6	0.74	0.54	Cluster_1
**ILC1302**	91.5	276.5	0.94	185	159.06	184	137.5	1.08	0.33	Cluster_1
**FLIP09-96C**	96.17	204	0.73	107.83	140.06	150.08	130.71	0.85	0.47	Cluster_1
**FLIP07-225C**	99	209.5	0.77	110.5	144.02	154.25	134.46	0.85	0.47	Cluster_1

**FLIP09-227C**	101.5	126	0.48	24.5	113.09	113.75	112.43	0.31	0.81	Cluster_2
**FLIP09-136C**	130.83	137.5	0.67	6.67	134.13	134.17	134.08	0.08	0.95	Cluster_2
**FLIP07-187C**	15.83	73.5	0.04	57.67	34.11	44.67	26.05	1.27	0.22	Cluster_3
**FLIP09-139C**	16.5	137.5	0.08	121	47.63	77	29.46	1.42	0.12	Cluster_3
**FLIP09-197C**	18	56	0.04	38	31.75	37	27.24	1.1	0.32	Cluster_3
**FLIP90-96**	20	149	0.11	129	54.59	84.5	35.27	1.4	0.13	Cluster_3
**FLIP07-218C**	21	91.5	0.07	70.5	43.83	56.25	34.16	1.24	0.23	Cluster_3
**FLIP07-227C**	22	148.5	0.12	126.5	57.16	85.25	38.32	1.38	0.15	Cluster_3
**FLIP09-102C**	23	102.5	0.09	79.5	48.55	62.75	37.57	1.25	0.22	Cluster_3
**FLIP07-221C**	36	107.5	0.14	71.5	62.21	71.75	53.94	1.07	0.33	Cluster_4
**Arifi**	36.5	150	0.2	113.5	73.99	93.25	58.71	1.22	0.24	Cluster_4
**FLIP08-82C**	41	105.5	0.16	64.5	65.77	73.25	59.05	0.99	0.39	Cluster_4
**FLIP09-290C**	41.5	115.5	0.18	74	69.23	78.5	61.06	1.03	0.36	Cluster_4
**FLIP09-135C**	43.5	134.5	0.22	91	76.49	89	65.74	1.09	0.32	Cluster_4
**Farihane**	50.17	169	0.32	118.83	92.08	109.58	77.37	1.14	0.3	Cluster_4
**FLIP09-304C**	50.5	165	0.31	114.5	91.28	107.75	77.33	1.12	0.31	Cluster_4
**Douyet**	52	167.5	0.32	115.5	93.33	109.75	79.36	1.11	0.31	Cluster_4
**FLIP07-209C**	57.5	139	0.3	81.5	89.4	98.25	81.35	0.95	0.41	Cluster_4
**FLIP07-184C**	59	165	0.36	106	98.67	112	86.92	1.04	0.36	Cluster_4
**ILC533**	60.5	125	0.28	64.5	86.96	92.75	81.54	0.83	0.48	Cluster_4
**FLIP08-84C**	68.5	139.5	0.36	71	97.75	104	91.88	0.82	0.49	Cluster_4

Ys, seed yield under combined Heat-drought stress; Yp, seed yield in normal condition; HI, Harvest index; GMP, geometric mean productivity; MP, mean productivity; TOL, tolerance index; STI, stress tolerance index; HARM, harmonic mean; SSI, Stress susceptibility index; YSI, Yield Stability Index. Cluster represents tolerance level of chickpea accessions (cluster1, tolerant genotypes; cluster2, moderately tolerant, cluster3, susceptible genotypes, cluster4, moderately susceptible).

Table S9: Chickpea genotypes identification

**Genotypes**	**Pedigree**
ILC263	PI 339223 (Suceptible Repeated Check)
ILC482	ACC. no .26780-68
ILC533	F 58 (Susceptible Repeated Check)
ILC1302	ICC 8262
ILC3397	Suceptible Repeated Check for leav miner
ILC12004	NEC 2861
FLIP90-96	X87TH26/ILC 5342xFLIP 84-93C
FLIP97-7	X94TH76/FLIP90-15XILC6118
FLIP04-5	X98TH68(FLIP93-24CXILC6119)XS96114
FLIP07-75	X03TH25/(S00834 XFLIP 98- 121C)CA9783009
FLIP07-218C	X03TH-154/FLIP 97-185CXFLIP98-200C
FLIP09-96C	X05TH90/FLIP81-293XFLIP00-17
FLIP09-135C	X05TH148/DZ-10-11XFLIP98-107
FLIP09-148C	X06TH6/X05TH103XFLIP03-120
FLIP09-229C	S00794(30 KR)-13
FLIP09-290C	X04TH147/FLIP00-17XFLIP98-230
FLIP07-209C	X03TH-129/FLIP98-131CXFLIP99-47C
FLIP07-227C	X02TH94/FLIP97-149C X Sel01ter 73605.

FLIP08-82C	X79 TH221/ILC 72 x ILC 1922
FLIP08-84C	X79 TH16/ILC 630 x ILC 200
FLIP09-136C	X05TH152/FLIP98-107XUC27
FLIP07-187C	X03TH-146/FLIP98-130CXFLIP 97-25C.
FLIP07-225C	X02TH69/S00792 X FLIP98-028C
FLIP07-184C	X03TH-146/FLIP98-130CXFLIP 97-25C.
FLIP07-211C	X03TH132/FLIP 97-185CXFLIP99-47C
FLIP07-221C	X03TH-159/FLIP98-131CXFLIP98-37C
FLIP09-139C	X05TH170/FLIP97-85XICCV-96030
FLIP09-102C	X05TH99/FLIP02-41CXFLIP97-85
FLIP09-111C	X05TH112/FLIP97-121XFLIP00-17
FLIP09-197C	X06TH57/FLIP97-110XFLIP02-36
FLIP09-301C	X04TH157/S01227XFLIP98-137
FLIP09-314C	X04TH185/ICC 12004XFLIP96-154
FLIP09-81C	X05TH53/X04TH-177XX04TH-178
FLIP09-274C	X04TH135/FLIP97-205XFLIP97-83
FLIP09-221C	S00789(30 KR)-7/
FLIP09-222C	S00789(30 KR)-19/
FLIP09-227C	S00789(30 KR)-40/
FLIP09-304C	X04TH159/S01230XFLIP98-230
Moubarak	X81 TH105/ILC 72 x ILC 484
Farihane	X80 TH176/ILC 72 x ILC 215
Douyet	X80 TH176/ILC 72 x ILC 215
Arifi	X95TH 2/FLIP91-18XFLIP90-96
Zahour	X80 TH176/ILC 72 x ILC 215
